# Physical Trace Gas Identification with the Photo Electron Ionization Spectrometer (PEIS)

**DOI:** 10.3390/s24041256

**Published:** 2024-02-16

**Authors:** Theodor Doll, Victor M. Fuenzalida, Helmut Schütte, Stefan Gaßmann, Juan J. Velasco-Velez, Robert Köhler, Alex Kontschev, Thomas Haas, Bert Ungethüm, Andreas Walte, Jonas Oberröhrmann, Adrian Onken, Kasimir M. Philipp, Minh-Hai Nguyen, Thomas Lenarz, Achim Walter Hassel, Wolfgang Viöl

**Affiliations:** 1 Biomaterial Engineering, ENT, Hannover Medical School, 30625 Hannover, Germany; onken.adrian@mh-hannover.de (A.O.); nguyen.minh-hai@mh-hannover.de (M.-H.N.); lenarz.thomas@mh-hannover.de (T.L.); 2 Laboratorio de Superficies y Nanomateriales, Departamento de Física, FCFM, Universidad de Chile, Av. Blanco Encalada 2008, Santiago de Chile 8370448, Chile; vfuenzal@ing.uchile.cl; 3 Department of Engineering, Jade University of Applied Sciences, 26389 Wilhelmshaven, Germany; helmut.schuette@jade-hs.de (H.S.); stefan.gassmann@jade-hs.de (S.G.); 4 ALBA Synchrotron Light Source, Cerdanyola del Valles, 08290 Barcelona, Spain; jvelasco@cells.es; 5 Faculty of Engineering and Health, University of Applied Sciences and Arts, Von-Ossietzky-Straße 99, 37085 Göttingen, Germany; robert.koehler@hawk.de (R.K.); wolfgang.vioel@hawk.de (W.V.); 6 Adlantis GmbH, 44263 Dortmund, Germany; alex.kontschev@gmx.de (A.K.); haas.thomas@gmx.net (T.H.); 7 Airsense Analytics GmbH, 19061 Schwerin, Germany; ungethuem@airsense.com (B.U.); walte@airsense.com (A.W.); 8 Eyyon/DBT GmbH, 97070 Wuerzburg, Germany; jo0198@freenet.de (J.O.); km.philipp@posteo.de (K.M.P.); 9 Institute of Chemical Technology of Inorganic Materials, Johannes Kepler University Linz, 4040 Linz, Austria; achimwalter.hassel@jku.at

**Keywords:** volatile organic compounds (VOCs) identification, nano-vacuum electronics, electron impact ionization, MEMS chemosensor, external photo effect

## Abstract

Chemosensor technology for trace gases in the air always aims to identify these compounds and then measure their concentrations. For identification, traceable methods are sparse and relate to large appliances such as mass spectrometers. We present a new method that uses the alternative traceable measurement of the ionization energies of trace gases in a way that can be miniaturized and energetically tuned. We investigate the achievable performance. Since tunable UV sources are not available for photoionization, we take a detour via impact ionization with electrons, which we generate using the photoelectric effect and bring to sharp, defined energies on a nanoscale in the air. Electron impact ionization is thus possible at air pressures of up to 900 hPa. The sensitivity of the process reaches 1 ppm and is equivalent to that of classic PID. With sharpened energy settings, substance identification is currently possible with an accuracy of 30 meV. We can largely explain the experimental observations with the known quantum mechanical models.

## 1. Introduction

Trace gases in the air determine many hazardous situations such as chemical accidents or affect our health not only passively but also actively via markers in medical diagnostics. To date, the identification of trace substances in the air has mainly been achieved using mass spectrometry (MS), in which the necessary information is determined via the mass/charge ratio of the ions. Ideally, the retention time is also used for identification in advance by means of gas chromatographic pre-separation (GCGC-MS). Ion mobility spectrometry (IMS), in which the drift time of the ions in the air is determined, can also be used for very low concentrations of the target substances. It has the advantage that the device does not require a high vacuum, but it also has the disadvantage that not all substances can be detected and there is no referencing. In addition, chemical reactions cannot be ruled out with ionization at atmospheric pressure. Photon ionization is also a method that has been used in IMS in recent years. Direct ionization steps have been used by means of vacuum UV sources, but indirect ionization steps, which are excited by two-photon processes using lasers, have also been used. The latter laser–IMS (LIMS) devices [1] can very selectively detect substances (usually aromatics) at very low concentrations in complex mixtures. LIMS or MS systems are relatively cost-intensive and almost not portable. However, there are hand-held IMS-based devices on the market, which are still larger in size than the solution sought here due to the required drift field.

Small hand-held measuring devices equipped with a photoionization detector (PID) and other micro gas sensors (e.g., metal oxide sensors [2] or electrochemical cells) have been available on the market for years. The latter micro gas sensors detect the presence of gas groups with considerable sensitivity but lack any referenceable gas identification. In the former PID, substances in the air are ionized using high-energy UV light, and the ion current to be measured is then proportional to the concentration of the substances. As a rule, gas discharge lamps filled with krypton are used, which deliver a photon energy of 10.6 eV. This means that a PID alone is also not suitable for identifying substances, as the detector only provides information about the presence of substances with an ionization potential (IP) of less than or equal to 10.6 eV. Pollutants such as carbon monoxide (14.01 eV), hydrogen cyanide (13.65 eV), chloropicrin (11.42 eV), 1,2-dichloroethane (11.12 eV), and formaldehyde (10.87 eV) cannot be detected with a PID. The only way to detect some of these substances is to use a different UV source with a higher energy lamp, such as the 11.7 eV argon discharge lamp [3]. The problem with that lamp is the hygroscopic UV window made of lithium fluoride, which only lasts around 6 weeks. Ideally, a UV lamp would be suitable, in which the wavelength could be varied to enable the identification of the substance via its ionization potential. This is currently only possible on a technically large scale using the synchrotron radiation of a ring accelerator.

Since the 2000s, attempts have been made to transfer the principle of the miniaturized photoionization detector (PID) to electron impact ionization in free air and to use this to create an ionizer with tunable energy [4,5]. The idea is reminiscent of the Franck–Hertz experiment [6] from 1914, which is now part of many laboratory practicals in university courses. However, the resonance curves measured there are so heavily smeared in terms of energy with half-value widths (FWHM) that no identification of approx. 3000 relevant substances/molecules, which are listed in the NIST database [7] with a typical 6–7 meV energy difference, could ever have been realized from the measurement data.

The main reason for the low line sharpness in the Franck–Hertz experiment is the use of thermal electrons and possible collisions of electrons with the gas molecules during acceleration, even though the collisions should be elastic below the resonance or ionization limits. Based on these considerations and previous work [8], the external photoelectric effect was chosen to generate free electrons in air. Here, it is possible to obtain higher energy levels with UV-LED wavelengths that are selected to match the emitter materials’ work function *Φ*. Compared with the year 2000, UV LEDs are now available with wavelengths of down to 250 nm (4.96 eV), typically have a spectral width of 10…20 nm, and reach an electrical power of 6 watts. As far as emitter materials are concerned, metallic materials are initially preferable as they have a high electron density at the filled valence band edge. In addition, due to the availability of LEDs, materials with the lowest possible work function were also a priority in the past, which is why experiments were carried out with lanthanum hexaboride (LaB_6_) and later samarium (Sm) and ytterbium (Yb). Unfortunately, all these materials react with air and air humidity. Sm reaches a lifetime of a few days, Yb loses its emissivity to air within hours [9].

LaB_6_ remains emissive but loses its low work function of ideally 2.31 eV to significantly higher values [10]. Subsequently, elements that naturally occur in the solid state were investigated and alloys thereof. Under different combinations of Ag, As, Ce, In, and Sm, As_x_Ce_y_Sm_z_ showed a comparatively high emissivity at a work function *Φ* of 1.87 eV and a stability of >14 months [5]. Unfortunately, the reproducible fabrication of alloys of high- and low-melting materials poses technical difficulties, so a simple silver layer was used for further experiments. This loses a large part of its emissivity within months due to the formation of sulfide layers but can be reconstituted by Ar-sputtering.

**Figure 1:**
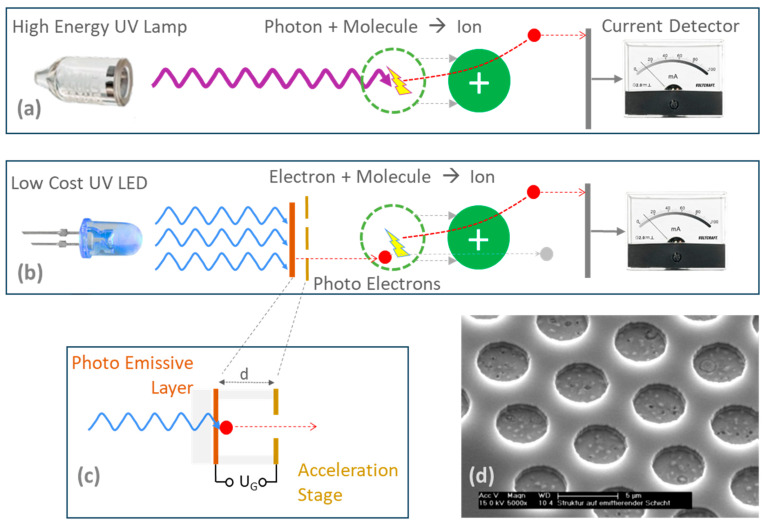
Derived from the principle of photoionization detectors PIDs (**a**), the photo electron ionization spectrometer PEIS (**b**) allows for tunable energies for the screening of ionization limits of trace gases. Tuning is achieved by an electronic acceleration stage (**c**) being fabricated as a thin-film array of 10 Mio emitters (**d**).

The acceleration structure was designed as a planar electrode on spin-on-glass or SU8 resist insulators and is shown in Figure 1d. The insulator thickness of 700 nm is approximately ten times the mean free path length of nitrogen–nitrogen collisions, 70 nm at normal conditions. At 100 hPa and below, however, the regime of nano-vacuum technology is already reached, i.e., collisions between accelerating electrons and air molecules are already unlikely. Molecules to be detected, such as hydrocarbons, have an even smaller impact cross-section due to the Ramsauer minimum and even slightly increase the mean free path [11].

During photoionization, an atom or molecule in a bound state /*i>* absorbs a photon of energy *hν*, exceeding the ionization energy *E_ion_*. If *hν > E_ion_* and an electron is lifted over the vacuum edge: the excess energy *hν* − *E_ion_* is thereby transformed into the kinetic energy of the ionization fragments. In electron impact ionization, in addition to this energy consideration, the momentum in the transition to the multibody system (electron plus molecule -> two electrons plus ion) must also be taken into account. In 1953, Wannier developed the basic theoretical description of the processes at the ionization threshold [12] and established the relationship

*σ*~(*E_el_* − *E_ion_*)^1.127^ (1)

for the ionization cross-section σ, where *E_el_* is the energy of the incident electron. For energies well above the ionization threshold, the ionization cross section increases rapidly and is better described by other models [13,14,15,16]. These are well summarized in the Lotz approximation [17] in agreement with experimental data. Whether resonances occur in the vicinity of the ionization threshold is still being discussed [18,19] and what this means for the idea of two electrons at rest next to an ion, especially in ambient air, is not clear.

In the following, we report on the first measurements with PEIS on various substances. In addition, we consider comparative measurements of PEIS with PID for an initial determination of the sensitivity of the method and test whether electron impact ionization can also be used to measure beyond the 10.6 eV limit. This is followed by PEIS measurements in spectrometer mode with a comparison of broad and sharper energy distributions—also with regard to previously reported, preliminary resonance-like results [20].

## 2. Materials and Methods

**Figure 2:**
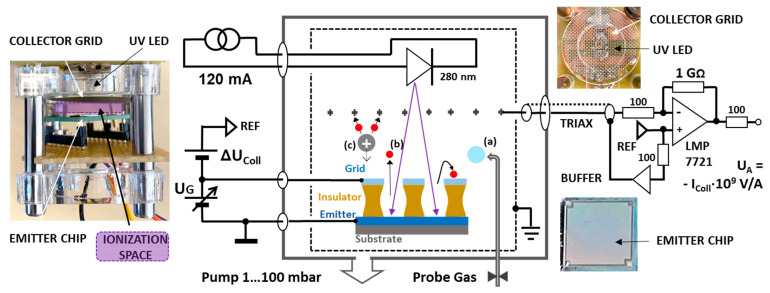
Testing setup with silver emitter layer chips: The middle section shows the shielded measurement chamber with the chip (not to scale) and the frontside illumination by the 280 nm UV-LED through the collector and accelerator grids, incoming gas species (a), generated photo electrons (b), and the ionization products (c). The left section shows a side view of the setup with an ionization space of 10 mm in height. On the right, front views of the collector grid and the UV LED behind are depicted as well as the actual thin-film emitter chip together with the fA-transconductance current amplifier schematic used (the Keithley 6517 B picoammeter uses a similar input circuit).

PEIS emitter chips of 10 × 10 mm^2^ size were fabricated on silicon substrates in thin film technology. The emitter materials were either LaB_6_ (pulsed laser deposition) with spin-on-glass insulators or Ag (50 nm thickness from sputtering) with SU8 resist insulators. The top acceleration grid was fabricated in aluminum (sputtered, 200 nm thickness), and the openings were lithographically defined and wet-chemically opened. Subsequent removal of the insulators was performed by conformal oxygen plasma etching to bare the emitter layers. These devices were used for frontside illumination with collector and accelerator grids. The collector grids were stainless steel and mounted approx. 10 mm above the PEIS chips (Figure 2).

For the LaB_6_ layer, a standard 10.6 eV PID-lamp was used in order to perform a comparison test with an industrial PID instrument (G 460 type, GfG Dortmund, Dortmund, Germany). The electronic instrument used to drive the PEIS chips and read the collector currents was a Keithley 4200 parameter analyzer with fA input amplifiers. For gas exposure, the PEIS was kept in a variable pressure chamber (5–900 hPa) connected to a cooled (0–15 °C) gas washing bottle containing the VOC liquids and additional dilution by purified normal air. The PEIS chips with the silver layer were illuminated by a 280 nm LED driven at 120 mA. Here, the readout instrument was either a transconductance amplifier, as shown in Figure 2, or a Keithley 6517 B picoammeter with an internal voltage source plus a RIGOL 832A power source for the variable grid voltage. The gas measurement setup was another pumped variable pressure chamber (0.5 … 100 hPa), and the diluted VOCs were delivered via a needle valve from the sample gas bags into which various solvents were injected by a microliter syringe.

## 3. Results

### 3.1. PEIS-PID Comparison

**Figure 3:**
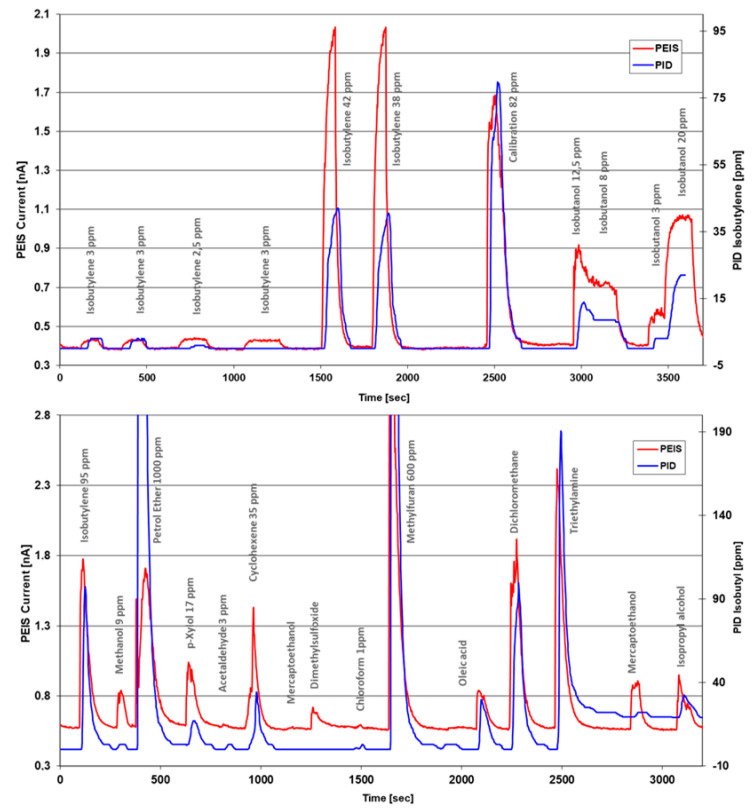
PID−PEIS comparison with isobutylene calibration gas (upper half) and the normalization of both signals. With the 82 ppm exposure, normalization of both signals was performed for the sensitivity testing of isobutanol and the series of substances shown in the lower row, where, after triethylamine, the PID baseline shifted.

In the first set of experiments, the LaB_6_ PEIS arrangement was used in parallel to the industrial PID sensor. Isobutylene from a gas bottle was applied to check the overall sensitivity, as depicted in Figure 3 (upper half). The red PEIS curve exceeds the readings of the PID sensor by 120%. With the 82 ppm peak to isobutylene, the sensitivity of the PEIS was normalized to that of the PID device, followed by isobutanol exposure, continued by the lower half of that figure. It shows the responses towards a series of substances: isobutylene, methanol, petrol ether, *p*-Xylene, acetaldehyde, mercaptoethanol, dimethyl sulfoxide, chloroform, oleic acid, methyl furane, and trimethylamine. At the end of this sequence, the PID sensor shifted its baseline, but the PEIS again showed stable responses to mercapto- and isopropyl alcohols. It is clearly observed that the calibration factors of both devices will vary, and the PEIS signals are more prominent, e.g., towards DMSO and *p*-Xylene.

### 3.2. First PEIS Spectrometry

**Figure 4:**
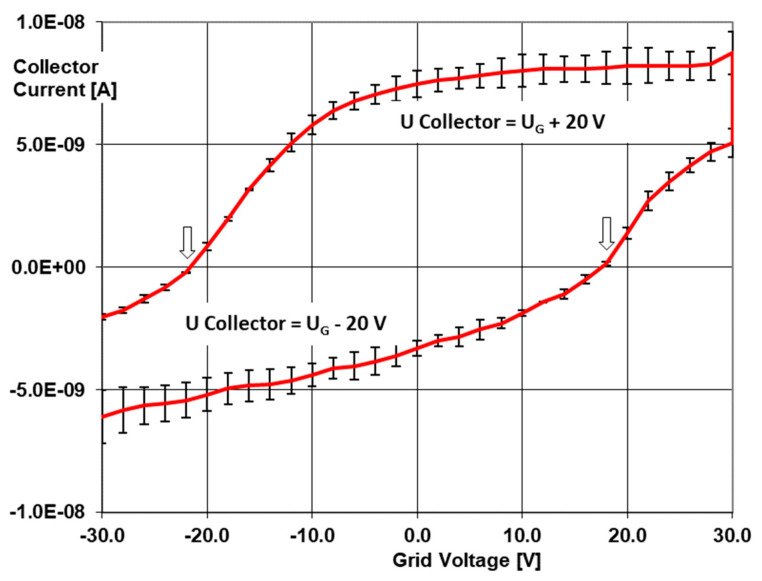
Photo electron ionization spectrogram of 2–mercaptoethanol (exemplary) at 5 hPa. The collector voltages are added to the grid voltage. Lower curve: repelling collector voltage; upper curve: further acceleration.

The LaB_6_ PEIS setup was then pumped down towards 5 hPa and used in its spectrometer mode with acceleration voltages varied from −30 V to +30 V and back in three cycles. The stepping width was 1 Volt, and multiple data points that were collected yielded error bars. Figure 4 shows exemplary data for 2-mercaptoethanol. Both parts of the curve correspond to a collector voltage running in parallel to the accelerator grid with −20 V (repelling the ionization reactants, lower part) and +20 V (further accelerating, upper part of the curve). Similar spectrograms were taken with trimethylamine, *p*-Xylene, ethyl acetate, methanol, 3methyl-1butanol, mercaptoethanol, and isopropanol at 5, 50, and 500 hPa. They are available in the data repository.

As the curves come with several kinks (Table 1), various models were tested to relate those spectrograms to the respective ionization energies. The best results were achieved by using the zero current transitions of the accelerating grid voltage *U_G_* (*I =* 0) (see arrows in Figure 4):

*e·U_G_* (*I_coll_* = *0*) + *E_ion_* = (*h·ν* − *Φ*) + *e* (*U_coll_* − *U_G_*) (2)

by using the highest PID lamp energy of *hν* = 10.6 eV and *Φ_LaB_*_6_ = 2.7 eV. “*e*” denotes the electron charge necessary to align voltages with electron energies. Though the standard deviations at the zero crossing are the lowest of all data points, the prediction accuracy of ionization energies remains as low as 460 meV.

In a third round of experiments, a silver layer was used for electron emission in the PEIS chips, and frontside illumination was performed with a 280 nm (+/−6 nm) UV LED. This should lead to a sharper energy distribution of the photo electrons compared with the previous arrangements. The spectrometric measurements were refined down to 10 mV steps (U_G_) and the collector voltage was reduced to U_G_ = +100 mV in order to virtually rule out any penetration of the electrical collector field into the emitter structure. In this arrangement, the measured collector currents are now only in the range of 100 fA to 160 pA. These currents drifted considerably without being attributable to heating of the arrangement or charging effects. Nevertheless, strong fluctuations in the collector current signal were observed at the ionization thresholds of the applied VOCs or air components. By a high-resolution experiment with 50,000 data points, these fluctuations could be correlated with the noise or flickering of the accelerating voltage U_G_ at the accuracy limit of the source of the measuring device, as specified by the manufacturer (U_G_, _eff_ = 2.6 mV or U_Step_ = 10 mV).

**Figure 5:**
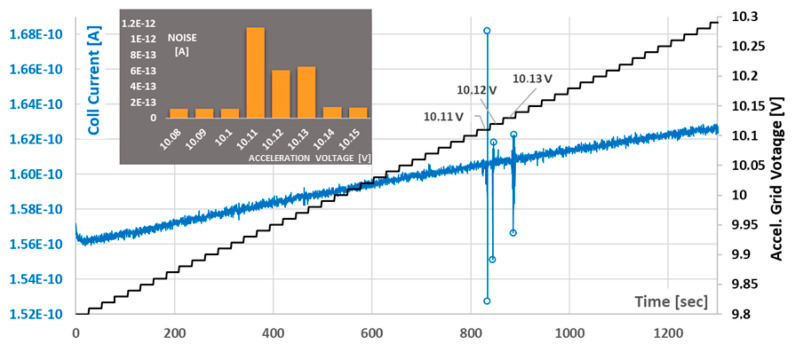
PEIS measurement of isopropyl alcohol (IPA) at 1 hPa from 9.8 eV to 10.3 eV with 10 meV steps (black). The collector current (blue) begins to flicker between 10.11 and 10.13 eV, which is close to the ionization energy of IPA (10.17 eV). The inset shows that the average noise (standard deviation) is a suitable tool for the search of such flickering events.

**Figure 6:**
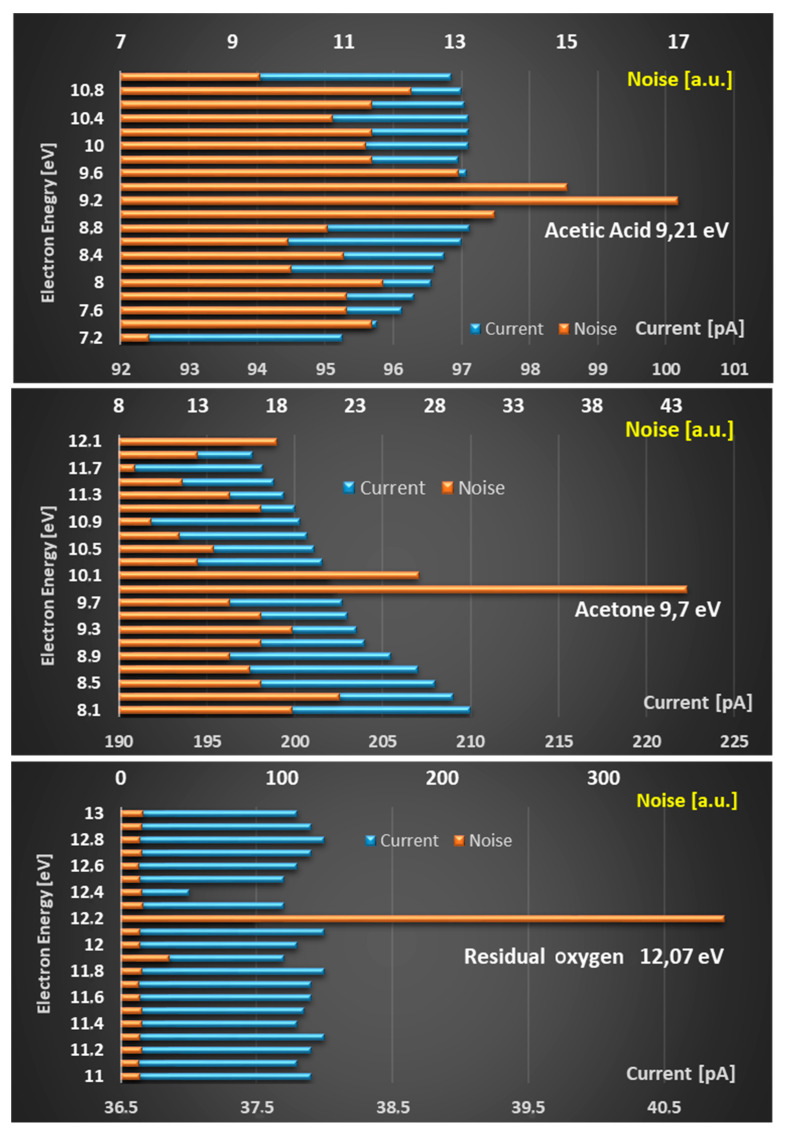
Steps towards PEIS substance identification via flickering search performed with 200 meV steps (acetic acid and acetone) and 100 meV steps (residual oxygen).

The fluctuations are only visible at the ionization thresholds. As a result, the measured signal oscillations were analyzed over their standard deviations (100 per measuring point) in parallel with the current measurements. The evaluation of such a measurement is shown in Figure 5: a flickering of the current signal becomes visible near the ionization threshold. As a result, the standard deviation rises sharply. It is noteworthy that as the ionization thresholds are clearly exceeded, the standard deviations decrease again, and the collector current signal does not make any noticeable jump either. These measurements via the standard deviations currently provide the best indications of an approach to the ionization energies of gases in PEIS arrangements. Figure 6 also shows such successful measurements for acetic acid, acetone, and residual oxygen, in each case, at 1 hPa. In view of the long measurement times for 100–200 measurement points per voltage step, coarser measurement steps of 100 meV and 200 meV were selected here.

## 4. Discussion

With regard to the comparison measurements with the LaB_6_ electron emitter/10.6 eV lamp and the PID sensor, the general equivalence is not surprising, as most of the substances could also be photoionized. However, the measurement with methanol (10.85 eV) and chloroform (11.5 eV) deserves attention. Methanol is seen relatively weakly by the PEIS and also by the PID with a very small deflection: this should not actually be present, as the 10.6 eV lamps emit at a wavelength range of 116.2–117.2 nm, which corresponds to a photon energy of 10.67–10.58 eV. However, the following is conceivable: As the PEIS and PID were arranged close to each other in the measurement and the PEIS structure was open, ions or electrons from the PEIS structure could have been captured by the PID, which could have led to a very small signal. The same applies to the tiny deflections at 1 ppm chloroform. The low concentration was chosen because the precipitation of this gas on the surfaces of the PEIS led to high surface currents at the insulators and falsified the measurement (in order to be able to measure 30 nA at 30 volts U_G_, an insulation resistance of 1 GΩ is required, which represents a technological challenge with 10 M emitter holes of 700 nm depth). Also, the test with oleic acid could be questioned, as oleic acid has a very low vapor pressure. Since only a technical grade material was used here, volatile impurities could have caused the signal.

The detection accuracy of only 470 meV determined in the first spectrometry tests may be due to the fact that the 10.6 eV lamps not only emit the 116.7 nm wavelength but also emit a larger 123.8 nm line (10.0 eV). This results in a systematic energy uncertainty of 600 meV in the ionization measurements, which matches the resolution limit. The extent to which the residual energy of the 10.6 eV photons is also sharply reflected in the kinetic electron energy after overcoming the work function, or whether the momentum cone of the Fermi sphere in question causes an additional energy broadening, still needs to be clarified.

In general, there are several possible explanations for the observed flickering of the PEIS measurements at the ionization thresholds. Firstly, it was necessary to check whether the multiplication of charge carriers in the ionization space (a cube of 1 cm in length) could possibly lead to space charge effects, as was the case with earlier electron tubes. According to a rough estimate, these are very unlikely due to the low excitation current of approx. 100 pA. In purely mathematical terms, at an acceleration voltage of 10 V, the electrons are traveling in the collision-free case at 1700 km/s, which is so fast that only about three electrons will be traveling at the same time. The space charge clouds in electron tubes are found at charge densities millions of times higher than this.

It also had to be investigated whether the measurement fluctuations in the experimental setup could actually achieve large fluctuations in the vicinity of the ionization thresholds. In principle, there are three simultaneously occurring reaction paths in the very narrow energy range around the ionization thresholds: the further travel of an electron without ionization, the loss of all electron energy at the threshold, and the continued movement of two electrons in the collector field if the threshold was clearly exceeded and kinetic residual energy is split. The energetic conditions are simulated in Figure 6. The LED spectrum with 280 +/− 6 nm is transferred to 4.43 eV center energy and a sigma ranging from 4.34 to 4.52 eV. All energies below the work function of polycrystalline silver are cut off from the overall spectrum due to the external photoelectric effect (left partial image). As the silver work function varies in the literature from 4.23 eV for photoemission from freshly prepared films [21] to 4.3 eV for contact potential measurements [22], a value of 4.26 eV is widely used and was taken here for calculations. Applying the grid voltage *U_G_* results in an electron energy distribution as shown in the right-hand partial image for *U_G_* = 10.03 V. One can see that the energy distribution is shifted to the right by

Δ = (*h*·*ν* − *Φ*) = 0.17 eV (3)

The interaction cross-section of the ionization starts at 10.17 eV in the Wannier and Lotz functionals for the IPA simulation example.

**Figure 7:**
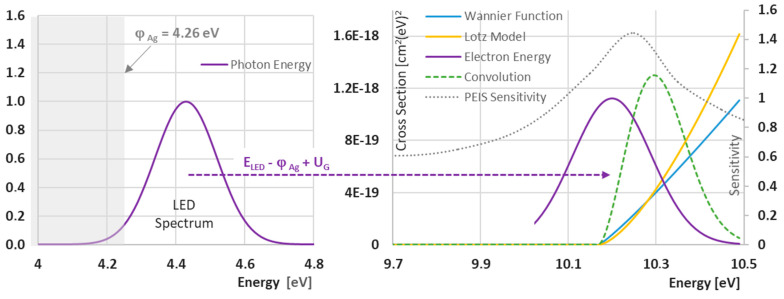
Modeling the flicker effect exemplified in the case of isopropyl alcohol (IPA): The part of the UV-LED spectrum that energetically exceeds the work function of the silver emitter (**left**) is mapped into a variable electron energy distribution. This is brought to the ionization limit of IPA (10.17 eV) by increasing the grid voltage. At this point, the Wannier function and the Lotz modeling take off (**right**). The convolution of the energy distribution with one of these functions can be transformed into a PEIS sensitivity function, which has a maximum near the ionization threshold. There, small fluctuations in *U_G_* cause very large output signal deviations, which can be apparent as flickering at the ionization threshold.

The convolution of the energy distribution function with the Lotz model provides a measure of the PEIS sensitivity to fluctuation in the measurement setup based on its relative increase (relative slope of the convolution divided by the input signal variable). The dotted curve in Figure 7 shows the development of this sensitivity, which would be obtained by varying *U_G_* in each case. Its maximum lies at 10.25 eV. The above considerations would result in an associated grid voltage of *U_G_* = 10.25 V *−* 0.17 V = 10.08 volts. Experimentally, the maximum of the noise was observed at 10.11 volts. The current resolution limit of the PEIS determination of the ionization energies would therefore be an achievable error of 30 meV and would be partly due to systematic reasons. The latter points to optimization possibilities. Firstly, however, the reliability of such flicker measurements would have to be increased.

In principle, there is a third consideration. If the ionization energy were hit sharply, two electrons with *E* = 0 would be obtained, i.e., they would remain next to their original ion. Readsorption would in turn lead to a frustrated Auger emission of an electron at rest. Slow drifts in the collector field would therefore have to be taken into account as well as possible resonances, as finally described by Franck–Hertz and later taken up again by Fano and Klar. The more precise quantum mechanical analysis of the processes, especially in the atmosphere, has so far eluded a simple conception and description.

## 5. Patents

T. Doll, H. Baum, DE102020113351A1, T. Doll, T. Haas, DE 10 2011 013 262 A1.

## Figures and Tables

**Table 1:** List of prominent points in the PEIS spectrograms of various VOC admixtures, measured at 5 hPa. The zero crossings are suitable for the approximate determination of ionization energies. However, the achievable resolution remains low.

		U_Collector_ = −20 V		U_Collector_ = +20 V			
Trace Gas	E_Ion_ [eV]	Zero Current [V]	1st Kink [V]	Zero Current [V]	1st Kink [V]	E_Ion_, Calc. [eV]	σ^2^ [e^2^V^2^]
Triethylamine	7.3	20.0	16	−18.5	3.0	7.15	0.02
p-Xylol	8.44	20.0	17	−19.0	10.0	7.40	1.08
Mercaptoethanol	9.65	17.5	15	−21.5	−9.0	9.90	0.06
Ethyl acetate	10.1	17.5	12.5	−22.0	5.0	10.15	0.00
3-Methyl-1-butanol	10.16	17.0	15	−22.0	−11.0	10.40	0.06
Isopropyl aclohol	10.17	17.3	14.5	−22.0	−12.0	10.25	0.01
Methyl alcohol	10.85	20.1	12.5	−27.0	6.0	11.35	0.25
						**rms [eV]**	**0.460**

## Data Availability

Dataset available on request from the authors.
